# Antagonistic and Detoxification Potentials of *Trichoderma* Isolates for Control of Zearalenone (ZEN) Producing *Fusarium graminearum*

**DOI:** 10.3389/fmicb.2017.02710

**Published:** 2018-01-18

**Authors:** Ye Tian, Yanglan Tan, Zheng Yan, Yucai Liao, Jie Chen, Marthe De Boevre, Sarah De Saeger, Aibo Wu

**Affiliations:** ^1^SIBS-UGENT-SJTU Joint Laboratory of Mycotoxin Research, Key Laboratory of Food Safety Research, Shanghai Institutes for Biological Sciences, University of Chinese Academy of Sciences, Chinese Academy of Sciences, Shanghai, China; ^2^College of Plant Science and Technology, Huazhong Agricultural University, Wuhan, China; ^3^Department of Resources and Environment Sciences, School of Agriculture and Biology, Shanghai Jiaotong University, Shanghai, China; ^4^Laboratory of Food Analysis, Department of Bioanalysis, Faculty of Pharmaceutical Sciences, Ghent University, Ghent, Belgium

**Keywords:** mycotoxins, zearalenone (ZEN), *Fusarium*, biological control, *Trichoderma*, modified mycotoxins

## Abstract

Fungi belonging to *Fusarium* genus can infect crops in the field and cause subsequent mycotoxin contamination, which leads to yield and quality losses of agricultural commodities. The mycotoxin zearalenone (ZEN) produced by several *Fusarium* species (such as *F. graminearum* and *F. culmorum*) is a commonly-detected contaminant in foodstuffs, posing a tremendous risk to food safety. Thus, different strategies have been studied to manage toxigenic pathogens and mycotoxin contamination. In recent years, biological control of toxigenic fungi is emerging as an environment-friendly strategy, while *Trichoderma* is a fungal genus with great antagonistic potentials for controlling mycotoxin producing pathogens. The primary objective of this study was to explore the potentials of selected *Trichoderma* isolates on ZEN-producing *F. graminearum*, and the second aim was to investigate the metabolic activity of different *Trichoderma* isolates on ZEN. Three tested *Trichoderma* isolates were proved to be potential candidates for control of ZEN producers. In addition, we reported the capacity of *Trichoderma* to convert ZEN into its reduced and sulfated forms for the first time, and provided evidences that the tested *Trichoderma* could not detoxify ZEN via glycosylation. This provides more insight in the interaction between ZEN-producing fungi and *Trichoderma* isolates.

## Introduction

**Figure 1:**
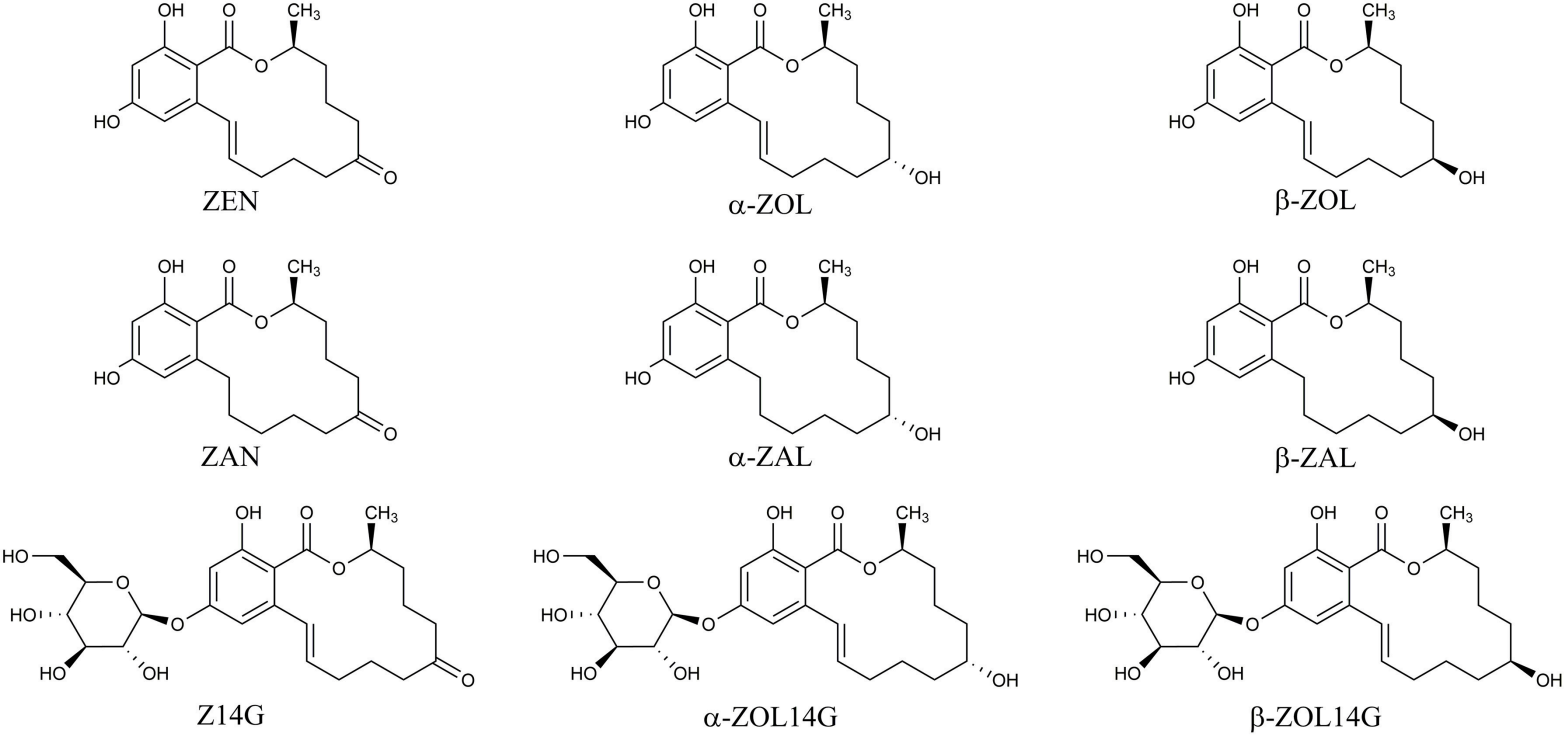
Chemical structures of ZEN, α-ZOL, β-ZOL, ZAN, α-ZAL, β-ZAL, Z14G, α-ZOL14G, and β-ZOL14G.

Mycotoxins are secondary metabolites produced by fungi with toxic effects on plants, animals and human (Hussein and Brasel, 2001). Among them, zearalenone (ZEN) is a mycotoxin with estrogenic potency, and commonly found in agricultural commodities globally (Nielsen et al., 2014). ZEN is mainly synthesized by a variety of *Fusarium* species, such as *F. graminearum, F. culmorum*, and *F. crookwellense*, which are plant pathogens capable of infecting crops and causing crop diseases in the field (Zinedine et al., 2007; Gromadzka et al., 2008). Besides ZEN, zearalanone (ZAN), α-zearalenol (α-ZOL), β-zearalenol (β-ZOL), α-zearalanol (α-ZAL), and β-zearalanol (β-ZAL) (Figure 1) are reduced derivatives of ZEN frequently detected in contaminated cereal grains or cultures of ZEN-producing *Fusarium* species (Zinedine et al., 2007). As toxic xenobiotics for plants, ZEN, α-ZOL, and β-ZOL can be bio-transformed into less toxic ZEN-14-glucoside (Z14G), α-ZOL-14-glucoside (α-ZOL14G) and β-ZOL-14-glucoside (β-ZOL14G) (Figure 1), which are common detoxification products in plant defense. Furthermore, some fungi also possess the capacity to convert ZEN into Z14G by conjugating endogenous glucose (Kamimura, 1986). The structures of these glycosylated mycotoxins were changed after modification, so they can't be detected by routine analysis, and they are termed as modified mycotoxins (Berthiller et al., 2013).

Both ZEN and its reduced forms are stable compounds, which exhibit hepatotoxic, hematotoxic, immunotoxic, and genotoxic effects. In addition, ZEN-related mycotoxins can competitively bind the estrogen receptors, causing alterations in genitals and reproduction disorders, posing a threat to human and animals health (Zinedine et al., 2007). To protect consumers, prevention before harvest seems to be an effective strategy for mycotoxin management (Jard et al., 2011; Tian et al., 2016a). Application of antagonistic biological control agents for controlling the toxigenic *Fusarium* spp., is a promising biological control based approach to reduce ZEN contamination. As potential antagonistic microbes, the genus *Trichoderma* has been widely studied for their capabilities against plant pathogenic fungi, and its biological control mechanisms mainly include faster growth speed and antibiotic production to compete nutrients and living space with pathogens, mycoparasitism mediated by producing cell wall degrading enzymes, and the ability to induce plant's defense system (Benítez et al., 2004; Sellamani et al., 2016; Tian et al., 2016a). Two *Trichoderma* isolates could effectively decrease the amount of mycotoxin ZEN produced by *Fusarium* spp. by a dual-culture assay (Gromadzka et al., 2009). In addition, other studies showed that some *Trichoderma* isolates also could inhibit mycotoxin deoxynivalenol (DON) production of *Fusarium* spp. (Busko et al., 2008; Matarese et al., 2012; Tian et al., 2016b). DON, a common type B trichothecene mycotoxin (Cuomo et al., 2007), usually co-occurs with ZEN in the foods and feeds (Molto et al., 1997; Castillo et al., 2002; Döll and Dänicke, 2011; Pietsch et al., 2013; Kovalsky Paris et al., 2014). Both DON and ZEN are frequently detected mycotoxins with high contamination levels (Stepien and Chełkowski, 2010). Recent work showed that DON could be bio-transformed into its modified form deoxynivalenol-3-glucoside (D3G) by *Trichoderma* isolates. D3G was generated in the defense of plants after infected by DON-producing pathogens, and D3G was regarded as a detoxification product of DON catalyzed by UDP-glucosyltransferases (Poppenberger et al., 2003; Schweiger et al., 2010; Li et al., 2015; Pasquet et al., 2016). Our recent study reported the occurrence of D3G in metabolism of selected *Trichoderma* isolates against DON producers (Tian et al., 2016b). However, little is known about the metabolism of ZEN in *Trichoderma* isolates. Thus, our particular interest was that whether *Trichoderma* spp. also possess the capacity to glycosylate ZEN into glycosylated forms for self-protection. Herein, the antagonistic potentials of *Trichoderma* isolates against ZEN-producing *F. graminearum* and the metabolism of ZEN in *Trichoderma* isolates was investigated in this work. A targeted LC-MS/MS method for simultaneous determination of ZEN and its reduced forms (α-ZOL, β-ZOL, α-ZAL, β-ZAL, and ZAN) and glycosylated forms (Z14G, α-ZOL14G, and β-ZOL14G) was applied to explore the anti-toxigenic activity of antagonists and ZEN metabolization in *Trichoderma* isolates. It was observed that three *Trichoderma* isolates could effectively suppress the mycelia spread and mycotoxin production of ZEN-producing *F. graminearum*. In addition, results of ZEN-treated experiment showed that the tested *Trichoderma* isolates could not detoxify ZEN via glycosylation, but could convert ZEN to its reduced (α-ZOL and β-ZOL) and sulfated metabolites (Z14S and ZOL14S). As far as we know, this is the first report of the metabolic activity of *Trichoderma* isolates on ZEN, which would provide more insights in the interaction between mycotoxin ZEN producing fungi and antagonistic *Trichoderma* isolates.

## Materials and methods

### Chemicals and reagents

The mycotoxin standards of ZEN, α-ZOL, β-ZOL, α-ZAL, β-ZAL, and ZAN were purchased from Sigma-Aldrich (St. Louis, MO, USA). The standards of Z14G, α-ZOL14G, and β-ZOL14G were kindly provided by the Laboratory of Food Analysis, Ghent University (Belgium). Methanol and acetonitrile (HPLC-grade) were purchased from Merck (Darmstadt, Germany). Ultrapure water (18.2 MΩ·cm) used in our experiments was obtained from a Milli-Q System (Bedford, MA, USA). Cleanert MC clean-up columns were purchased from Bonna-Agela Technologies (Tianjin, China). Other chemicals were obtained from Aladdin (Shanghai, China).

### Fungal isolates

ZEN-producing *F. graminearum* species were from the Huazhong Agricultural University. Eight *Trichoderma* isolates were used in this study: *T. harzianum* JF309, *T. koningii* GIM3.137, *T. harzianum* GIM3.442, *T. longibranchiatum* GIM3.534, *T. harzianum* Q710613, *T. atroviride* Q710251, *T. asperellum* Q710682 and *T. virens* Q710925. All these *Trichoderma* isolates could convert DON into D3G, as reported in our previous work (Tian et al., 2016b).

### Antagonistic potentials of *Trichoderma* isolates on growth and mycotoxin production of *F. graminearum* F1

The dual-culture test was performed to examine the antagonistic potentials of *Trichoderma* isolates for controlling ZEN-producing *F. graminearum* as described before (Matarese et al., 2012; Tian et al., 2016b). The mycelial disks (*F. graminearum* and *Trichoderma* spp. combinations) from actively-growing colonies were placed on a 9-cm diameter dish. In addition, a disk of *F. graminearum* was placed without disks of *Trichoderma* isolates (control). The *Fusarium*-*Trichoderma* combinations, as well as the controls were incubated at 25°C for 2 weeks. To evaluate the inhibition efficacy of *Trichoderma* isolates on mycelia growth of *F. graminearum* F1, the radius of each *F. graminearum* colony was measured to create growth curve as described in Matarese et al. (2012), and then the data were subjected to analysis of variance of regression to compare the slope of growth curves of the pathogen in the presence/absence of tested *Trichoderma* isolates.

### Treatment of *Trichoderma* isolates with ZEN

The tested *Trichoderma* isolates were activated on PDA medium at 25°C. Then, a mycelial disk of each activated *Trichoderma* strain was moved from the edge of the colony, and inoculated in a new dish containing of 10 ml PDA medium mixed with ZEN at different concentrations (0, 0.5, 1, 2, and 4 μg/ml). Pure ZEN was added into the PDA medium as described before (Utermark and Karlovsky, 2007). The mycelial disk of *Trichoderma* isolates placed on PDA medium without mycotoxin was as control. The dishes were incubated at 25°C, and growth radius of the tested *Trichoderma* isolates were measured two times a day until the mycelia of tested strains spread over the whole dish. Regression analysis of the growth data was performed to evaluate the inhibitory effects of ZEN on mycelial growth of *Trichoderma* isolates.

### Mycotoxin extraction

After incubation, the PDA medium and mycelia in the dish were dried and ground into powder for preparation, followed by adding 10 ml of ACN/water/formic acid (84/15.9/0.1, v/v/v) solution. The mixture was then shaken for 10 min, and subjected to ultrasonication for 30 min. Next the mixture was centrifuged at 4,000 rpm for 30 min. 1 ml of the supernatant was passed through a Cleanert MC column for clean-up by following the manufacturer instructions. Thereafter, the purified mixture was moved into a new tube, and evaporated to dryness by nitrogen gas at 45°C. Finally, the residue was re-dissolved with 1 mL of methanol/water (1/1, v/v) and filtered through a 0.22-μm filter for LC-MS/MS analysis.

### Mycotoxin analysis by LC-MS/MS

Mycotoxins were determined on an Accela 1250 UPLC system (Thermo Fisher Scientific, San Jose, CA, USA) coupled to a TSQ VantageTM (Thermo Fisher Scientific, San Jose, CA, USA) triple stage quadrupole mass spectrometer. Separation was performed on an Agilent Extend-C18 column (100 mm × 4.6 mm, 3.5 μm) at 30°C with a flow rate of 350 μL/min. The mobile phase consisted of water containing 5 mM ammonium acetate (A) and methanol (B). The gradient was as follows: 0 min 20% B, 1 min 20% B, 2 min 50% B, 8 min 100% B, 10 min 100% B, 13 min 20% B, 15 min 20% B. The injection volume was 10 μL.

For MS/MS analysis, the parameters were set as follows: interface voltage of 2.5 kV (ESI^−^); desolvation temperature of 270°C; nebulizing gas (N_2_) pressure of 50 psi and drying gas (N_2_) pressure of 25 psi; heat block temperature of 300°C. The quantitation and identification of target mycotoxins were performed in selected reaction monitoring (SRM) mode. The optimized MS/MS parameters for each analyte are listed in Table 1. Xcalibur™ software (Thermo Fisher Scientific, San Jose, CA, USA, 2011) was used for data processing.

**Table 1:** MS/MS parameters for detected mycotoxins in SRM mode.

**Mycotoxin**	**Precursor ion (m/z)**	**Retention time (min)**	**Product ion for quantification (m/z)**	**Collision energy (ev)**	**Product ion for identification (m/z)**	**Collision energy (ev)**
ZEN	317.2 [M-H]^−^	8.60	175.5	26	131.5	32
ZAN	319.1 [M-H]^−^	8.44	205.5	23	160.5	24
α-ZOL	319.3 [M-H]^−^	8.29	160.5	22	130.5	33
β-ZOL	319.3 [M-H]^−^	7.73	160.5	22	130.5	33
α-ZAL	321.3 [M-H]^−^	8.11	277.5	24	303.5	23
β-ZAL	321.3 [M-H]^−^	7.45	277.5	23	303.5	24
Z14G	479.0 [M-H]^−^	6.78	317.5	20	175.6	45
α-ZOL14G	481.0 [M-H]^−^	6.61	319.6	18	275.5	37
β-ZOL14G	481.0 [M-H]^−^	5.78	319.6	23	275.5	36

### Liquid chromatography high resolution mass spectrometry (LC-HRMS) analysis of ZEN bio-transformation products

LC-HRMS analysis was conducted on a UHPLC system (1290 series, Agilent Technologies, Santa Clara, CA, USA) coupled to a quadruple time-of-flight (Q-TOF) mass spectrometer (Agilent 6530 Q-TOF, Agilent Technologies, Santa Clara, CA, USA). Chromatographic separation was performed on the Agilent Extend-C18 column. The mobile phase consisted of water containing 5 mM ammonium acetate (A) and methanol (B), and the gradient elution program was: 0 min 20% B, 1 min 20% B, 2 min 50% B, 8 min 100% B, 10 min 100% B, 13 min 20% B, 15 min 20% B.

The parameters of HRMS were set as follows: sheath gas (N2) temperature, 350°C, sheath gas flow, 11 L/min; dry gas (N2) temperature, 350°C, dry gas flow, 11 L/min; capillary voltage, 3.8 kV in negative mode; fragmentor, 130 V; and nebulizer pressure, 40 psi. Agilent software MassHunter B.06.00 (Agilent Technologies, Santa Clara, CA, USA, 2012) was used for data processing.

### Statistical analysis

All the experiments were set up in triplicates. Data were presented as the mean ± standard error of the mean (SEM), and data were subjected to two-tailed student's *t*-test analysis or regression analysis with Graphpad Prism 5.01 (GraphPad Software, San Diego, CA, USA, 2007).

## Results

### Effect of *Trichoderma* isolates on growth of ZEN-producing *F. graminearum* F1 in co-culture assay

**Figure 2:**
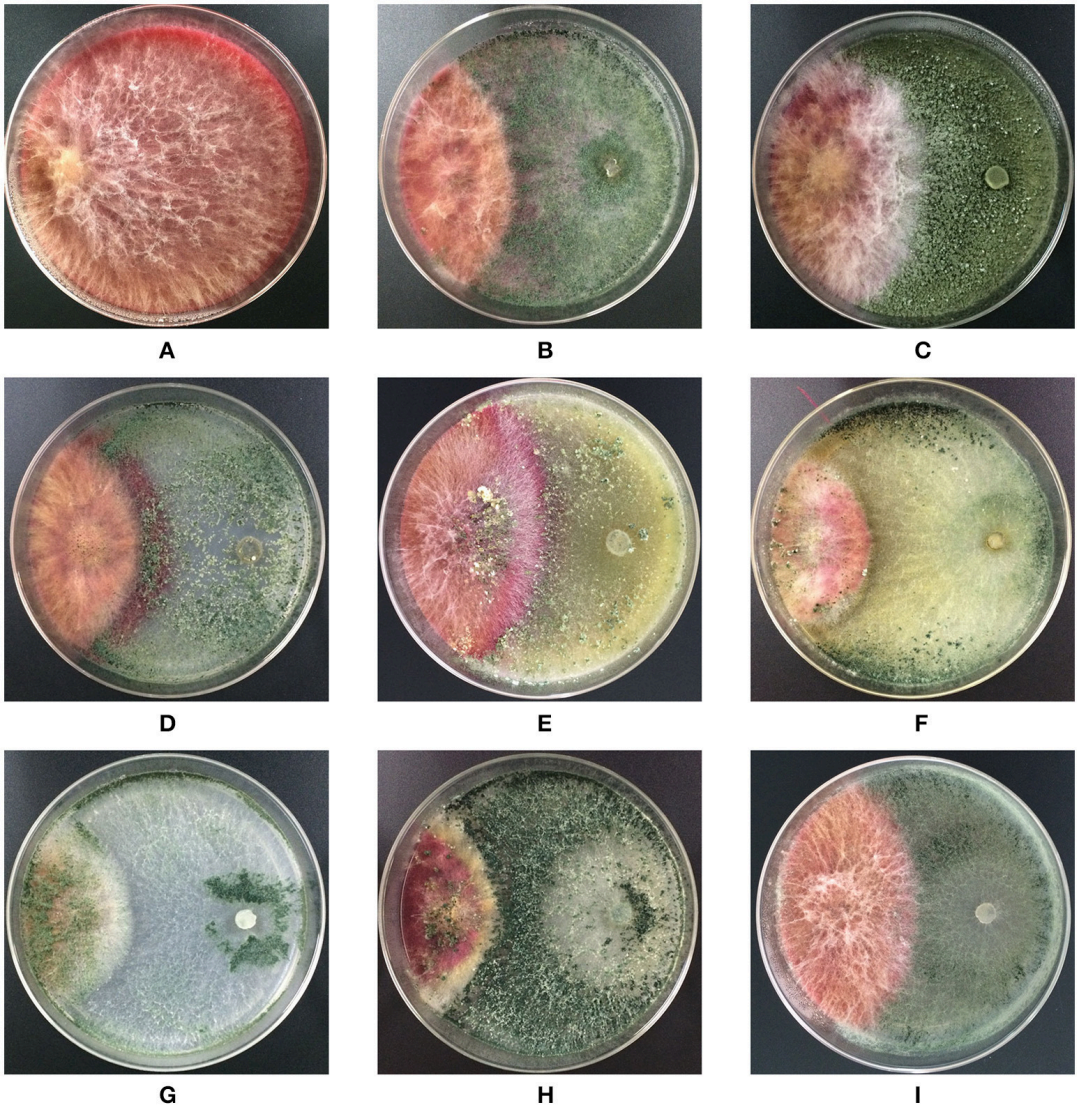
Colony morphology of *F. graminearum* F1 in co-culture assay after incubation on the potato dextrose agar (PDA). *F. graminearum* F1 grew alone **(A)**; *F. graminearum* 5035 grew against *T. harzianum* JF309 **(B)**; *T. koningii* GIM3.137 **(C)**; *T. harzianum* GIM3.442 **(D)**; *T. longibranchiatum* GIM3.534 **(E)**; *T. harzianum* Q710613 **(F)**; *T. atroviride* Q710251 **(G)**; *T. asperellum* Q710682 **(H)**; and *T. virens* Q710925 **(I)**.

*Trichoderma* isolates and *F. graminearum* F1 were co-cultured on PDA medium. These antagonists rapidly occupied the living space, and inhibited the mycelial spread of *F. graminearum* F1 due to their antagonistic activities (Figure 2). Growth inhibition is the main pattern for antagonists to manage the pathogen, and we found that seven of the tested *Trichoderma* isolates were able to significantly suppress the mycelial growth of *F. graminearum* F1 (Table 2). Furthermore, *T. harzianum* Q710613, *T. atroviride* Q710251 and *T. asperellum* Q710682 displayed more effective inhibitory effects, as we observed that these three *Trichoderma* isolates overgrew the colony of *F. graminearum* F1, and the mycelium of *Fusarium* in these pathogen-antagonist combinations were restricted to extend vertically (Figure 2). These results showed that *T. harzianum* Q710613, *T. atroviride* Q710251, and *T. asperellum* Q710682 were more effective suppressors for controlling the mycelia growth of *F. graminearum* F1.

**Table 2:** The inhibitory effect of antagonistic *Trichoderma* isolates on mycelial growth of *F. graminearum* F1 in dual culture.

**Combination**		**Regression parameters of growth curves**		
		***a***	***P***	***P slope***
FG F1 vs. *T. harzianum* JF309	Gc	0.49	<0.0001	<0.0001
	Gt	0.37	<0.0001	
FG F1 vs. *T. koningii* GIM3.137	Gc	0.49	<0.0001	NS
	Gt	0.45	<0.0001	
FG F1 vs. *T. harzianum* GIM3.442	Gc	0.49	<0.0001	<0.0001
	Gt	0.40	<0.0001	
FG F1 vs. *T. longibranchiatum* GIM3.534	Gc	0.49	<0.0001	<0.0001
	Gt	0.42	<0.0001	
FG F1 vs. *T. harzianum* Q710613	Gc	0.49	<0.0001	<0.0001
	Gt	0.35	<0.0001	
FG F1 vs. *T. atroviride* Q710251	Gc	0.49	<0.0001	<0.0001
	Gt	0.33	<0.0001	
FG F1 vs. *T. asperellum* Q710682	Gc	0.49	<0.0001	<0.0001
	Gt	0.32	<0.0001	
FG F1 vs. *T. virens* Q710925	Gc	0.49	<0.0001	<0.0001
	Gt	0.40	<0.0001	

*The radial growth rate of F. graminearum F1 facing antagonists on PDA medium compared with the radial growth rate of the control*.

*a, slope of the growth curve of F. graminearum (growth rate, mm/hour)*.

*P, significance of regression line*.

*P slope, significance of the difference between slopes of the pathogen F1 in the presence (Gt) and absence (Gc) of tested Trichoderma isolates*.

*NS, no significant difference*.

### Effect of *Trichoderma* isolates on mycotoxin production of ZEN-producing *F. graminearum* F1 in co-culture assay

To investigate the effect of *Trichoderma* isolates on ZEN-related mycotoxins production of *F. graminearum*, and verify whether *Trichoderma* could glycosylate ZEN into glycosylated forms. ZEN and its reduced derivatives (α-ZOL, β-ZOL, α-ZAL, β-ZAL, and ZAN), as well as three glycosylated mycotoxins (Z14G, α-ZOL14G, and β-ZOL14G), were monitored in this work.

**Figure 3:**
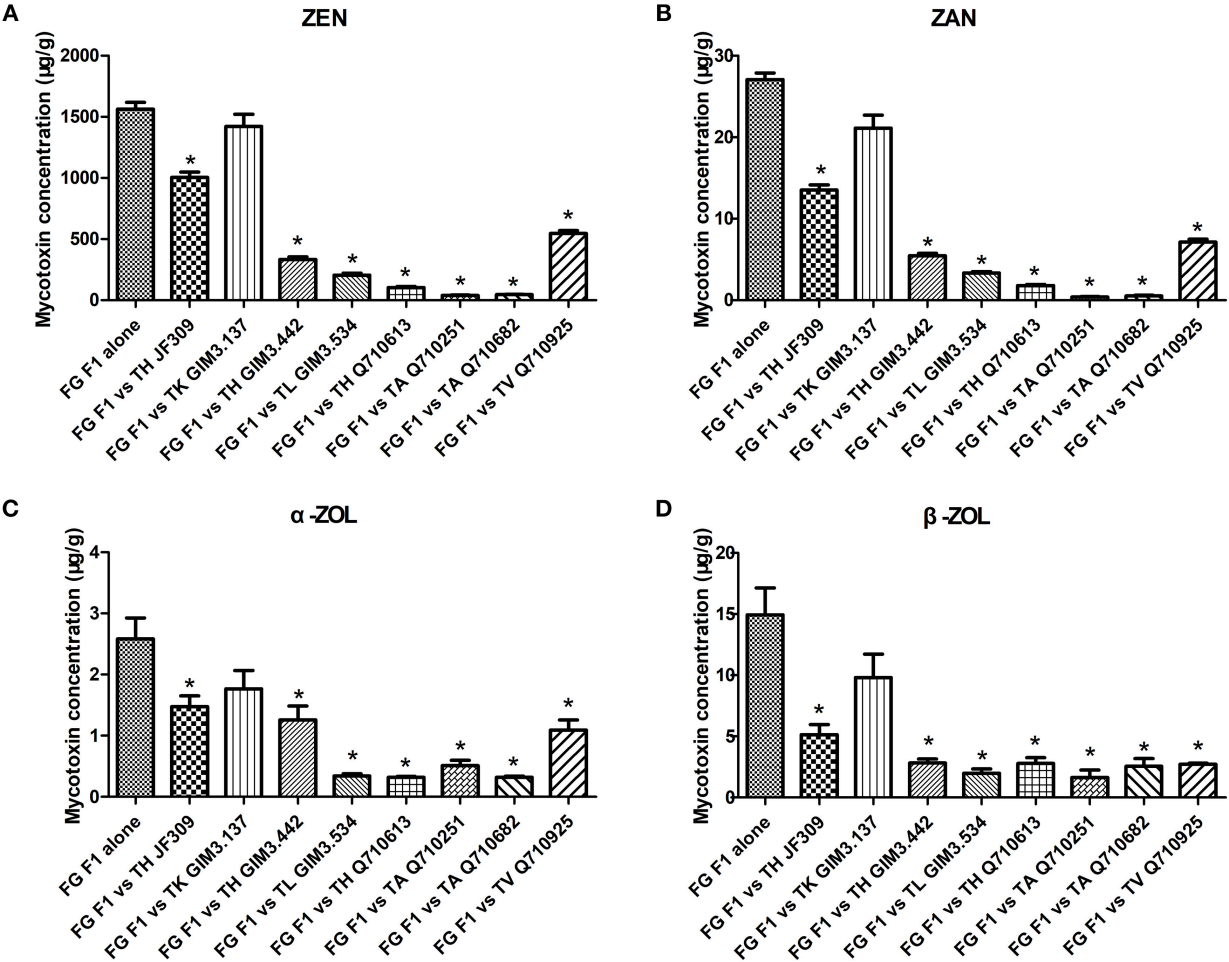
The inhibitory effect of antagonistic *Trichoderma* isolates on mycotoxin production (ZEN, **A**; ZAN, **B**; α-ZOL, **C**; and β-ZOL, **D**) of *F. graminearum* F1 in co-culture assay. From left to right: *F. graminearum* F1 grew alone, and grew against *T. harzianum* JF309, *T. koningii* GIM3.137, *T. harzianum* GIM3.442, *T. longibranchiatum* GIM3.534, *T. harzianum* Q710613, *T. atroviride* Q710251, *T. asperellum* Q710682, and *T. virens* Q710925. ^*^P < 0.05, significantly different from control.

*F. graminearum* F1 used in this dual-culture assay could produce 1562 μg/g ZEN, 27 μg/g ZAN, 2.6 μg/g α-ZOL, and 15 μg/g β-ZOL on PDA medium (Figure 3). ZEN was the major mycotoxin produced by the tested *F. graminearum*. When *F. graminearum* F1 grew against antagonistic *Trichoderma* isolates, the amount of mycotoxins produced by *F. graminearum* F1 was inhibited because of the antagonistic activity of *Trichoderma*. The inhibition rate of ZEN production ranged from 9 to 97%, for ZAN ranged from 22 to 98%, for α-ZOL ranged from 31 to 87%, and for β-ZOL ranged from 34 to 89%. Among the tested isolates, *T. koningii* GIM3.137 exhibited weaker inhibitory effects on the mycotoxin production of *F. graminearum* F1 (Figure 3). While *T. harzianum* Q710613, *T. atroviride* Q710251, and *T. asperellum* Q710682 exhibited a better efficiency in inhibiting mycotoxin production of *Fusarium*. When co-cultured with these three isolates, the amount of ZEN and ZAN produced by *F. graminearum* F1 was inhibited by over 93%, and the amount of α-ZOL and β-ZOL produced by *F. graminearum* F1 was inhibited by over 80%.

Unexpectedly, no glycosylated forms of ZEN and ZOL were observed when *Trichoderma* isolates were co-cultured with ZEN-producing *Fusarium*. The experiment, as described below, pinpointed the treatment of *Trichoderma* isolates with ZEN, and analyzed the metabolites to confirm the obtained result.

### Analysis of the metabolites when *Trichoderma* grew on PDA medium amended with pure mycotoxin ZEN

#### The inhibition of ZEN on growth of *Trichoderma*

**Figure 4:**
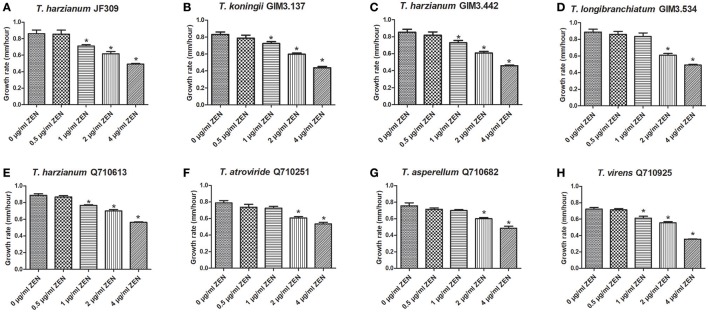
The effects of ZEN on mycelial growth of *Trichoderma* isolates: *T. harzianum* JF309 **(A)**, *T. koningii* GIM3.137 **(B)**, *T. harzianum* GIM3.442 **(C)**, *T. longibranchiatum* GIM3.534 **(D)**, *T. harzianum* Q710613 **(E)**, *T. atroviride* Q710251 **(F)**, *T. asperellum* Q710682 **(G)**, and *T. virens* Q710925 **(H)**. The tested *Trichoderma* isolates were inoculated on PDA amended with ZEN at different concentrations (0.5, 1, 2, and 4 μg/ml), and the control was inoculated on PDA without ZEN. ^*^P < 0.01, significantly different from control.

It has been reported that ZEN could inhibit the growth of some filamentous fungi, which help ZEN-producing *Fusarium* species compete with other microbes, so ZEN is regarded as a contributive factor for ZEN-producers (Utermark and Karlovsky, 2007). Firstly, we evaluated the inhibitory effects of ZEN on growth of various *Trichoderma* isolates, as the toxic effects of ZEN have not yet been elucidated in the genus *Trichoderma*. These effects were evaluated by comparing the mycelia growth rate of *Trichoderma* spp. when exposed to different ZEN concentrations (0, 0.5, 1, 2, and 4 μg/ml) on PDA medium. Results demonstrated that ZEN exhibited significant fungal toxic effects on *Trichoderma* isolates: the mycelia growth of five isolates were significantly inhibited by 1 μg/ml of ZEN. While for *T. longibranchiatum* GIM3.534, *T. atroviride* Q710251 and *T. asperellum* Q710682, the inhibitory effects were observed when treated with 2 μg/ml of ZEN (Figure 4).

Subsequently, all the *Trichoderma* isolates treated with 2 μg/ml ZEN were selected for further study of the metabolic activity of *Trichoderma* isolates on ZEN. The whole medium and mycelia were collected and prepared for analysis.

#### Analysis of the metabolites by LC-MS/MS

**Figure 5:**
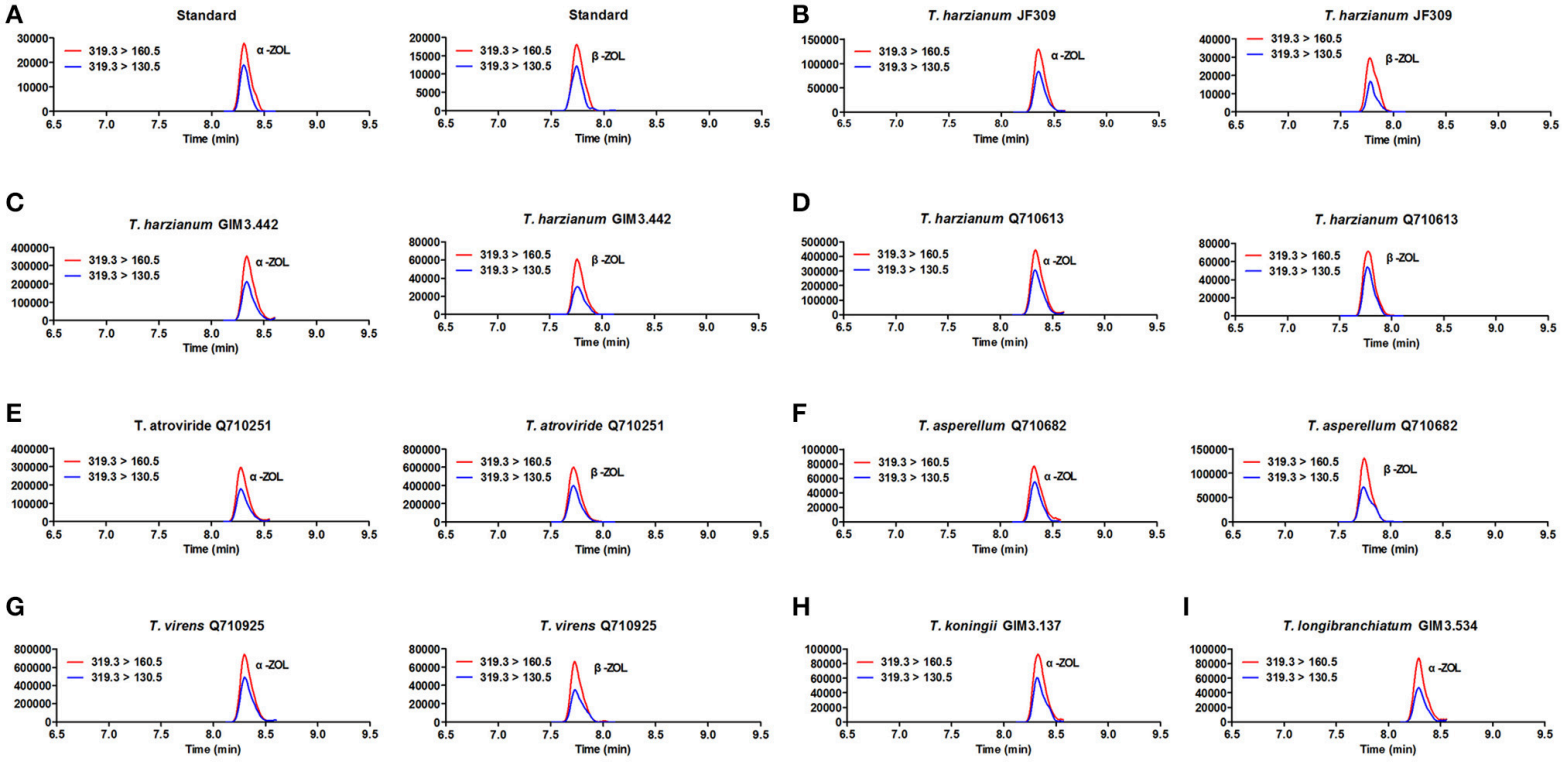
SRM chromatograms of α-ZOL and β-ZOL in standard solution (10 ng/ml) **(A)** and in *Trichoderma* samples after 2 μg/ml ZEN treatment **(B–I)**. The red line (m/z 319.3 > m/z 160.5) and blue line (m/z 319.3 > m/z 160.5) represent SRM traces for α-ZOL and β-ZOL, and the chromatographic retention time was used to distinguish the two ZOL isomers.

**Figure 6:**
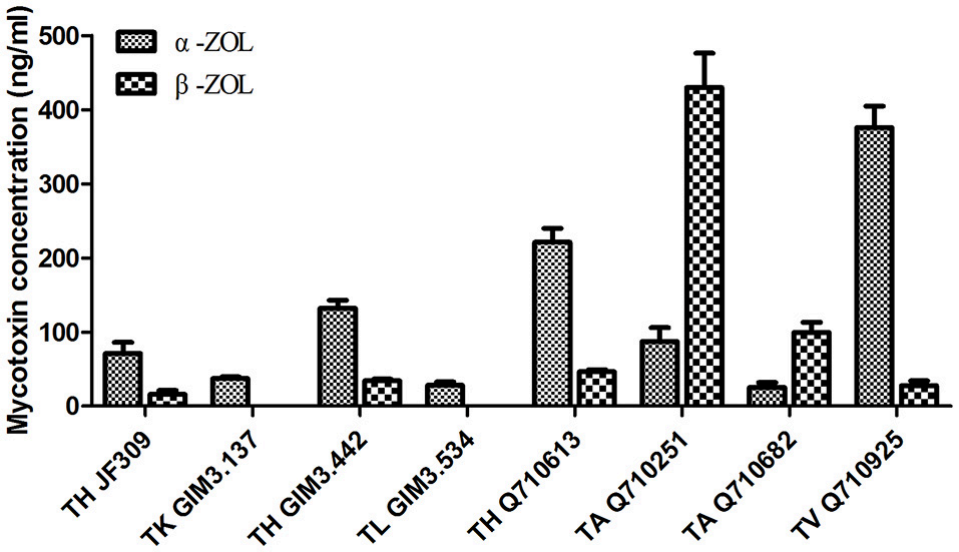
Concentrations of the metabolites (α-ZOL and β-ZOL) detected in samples of 2 μg/ml ZEN-treated *Trichoderma* isolates on PDA medium.

The results revealed that glycosylated mycotoxins (Z14G, α-ZOL14G, and β-ZOL14G) were not detected when *Trichoderma* isolates were cultured with ZEN. This was in accordance with the results of dual-culture assay. Interestingly, the reduced forms of ZEN (α-ZOL and β-ZOL) were detected in the samples (Figure 5). *T. harzianum* JF309, *T. harzianum* GIM3.442, *T. harzianum* Q710613, *T. atroviride* Q710251, *T. asperellum* Q710682, and *T. virens* Q710925 could metabolized ZEN into α-ZOL and β-ZOL (Figures 5B–G). Among them, *T. harzianum* JF309, *T. harzianum* GIM3.442, *T. virens* Q710925 converted more α-ZOL than β-ZOL, while *T. atroviride* Q710251 and *T. asperellum* Q710682 converted more β-ZOL than α-ZOL (Figure 6). For *T. koningii* GIM3.137 and *T. longibranchiatum* GIM3.534, only α-ZOL was observed in the ZEN-treated experiment (Figures 5H,I).

#### Analysis of metabolites by LC-HRMS

Besides glycosylation, sulfation is another detoxification process for different mycotoxins in plants and fungi. Zearalenone-14-sulfate (Z14S) was found to be a metabolite in *Arabidopsis thaliana, Rhizopus* spp. and *Aspergillus* spp. when exposed to ZEN, and zearalenol-14-sulfate (ZOL14S) was observed as a fungal metabolite in ZEN-treated trial (Berthiller et al., 2007; Brodehl et al., 2014). However, there were no reference standards (Z14S and ZOL14S) available for quantitative analysis by LC-MS/MS. For detection of sulfated forms of ZEN in prepared samples of *Trichoderma* treated with 2 μg/ml ZEN, a targeted method for screening modified mycotoxins was applied on the basis of LC-HRMS (Righetti et al., 2016). The negative precursor ions m/z 317.1394, 319.1551, 397.0963, and 399.1119 (theoretical m/z of [ZEN-H]^−^, [ZOL-H]^−^, [Z14S-H]^−^, and [ZOL14S-H]^−^, respectively) were mass-isolated by the quadruple mass filter, and then dissociated and detected by the TOF analyzer. The precursor ion m/z 397.0963 was observed in all samples and dissociated into a fragment of 317.14 which corresponds to the m/z of [ZEN-H]^−^. In the sample of *T. asperellum* Q710682, precursor ion m/z 399.1119 was found, and could yield a fragment of m/z 319.16 which corresponds to the m/z of [ZOL-H]^−^ (Table 3). This could be explained by the fact that sulfated metabolites yield fragments [Z14S-SO_3_-H]^−^ and [ZOL14S-SO_3_-H]^−^ after losing a sulfonic group (${\text{SO}}_{3}^{-}$). In addition, the other major fragments of precursor ions m/z 397.0963 and 399.1119 were in agreement with [ZEN-H]^−^ (m/z, 317.1394) and [ZOL-H]^−^ (m/z, 319.1551), respectively (Table 3). The fragments m/z 317.14, 175.07 and 131.09 for [Z14S-H]^−^ and the fragments m/z 319.15, 275.20, and 174.12 for [ZOL14S-H]^−^ were also reported before (Brodehl et al., 2014; Binder et al., 2017). In conclusion, these results revealed the presence of Z14S and ZOL14S in *Trichoderma* metabolism with ZEN treatment. For the first time, we reported that antagonistic *Trichoderma* isolates possess the detoxification capability to sulfate ZEN, and these sulfated forms would be quantified when reference standards are available in future.

**Table 3:** Summary of metabolites in samples of ZEN-treated *Trichoderma* isolates on PDA medium analyzed by LC-HRMS.

	**Target compound**	**m/z [M-H]**^**−**^		**Major fragments**
		**Theoretical m/z**	**Observed m/z**	
Mycotoxin standard	ZEN	317.1394	317.1386	289.15, 273.16, 175.07, 149.10, 131.09
	α-ZOL, β-ZOL	319.1551	319.1547	291.21, 275.20, 257.26, 174.12
*T. harzianum* JF309	Z14S	397.0963	397.0953	317.14, 289.15, 273.16, 175.07, 149.10, 131.09
	ZOL14S	399.1119	ND	ND
*T. koningii* GIM3.137	Z14S	397.0963	397.0949	317.14, 289.15, 273.16, 175.07, 149.10, 131.09
	ZOL14S	399.1119	ND	ND
*T. harzianum* GIM3.442	Z14S	397.0963	397.0954	317.14, 289.15, 273.16, 175.07, 149.10, 131.09
	ZOL14S	399.1119	ND	ND
*T. longibranchiatum* GIM3.534	Z14S	397.0963	397.0948	317.14, 289.15, 273.16, 175.07, 149.10, 131.09
	ZOL14S	399.1119	ND	ND
*T. harzianum* Q710613	Z14S	397.0963	397.0950	317.14, 289.15, 273.16, 175.07, 149.10, 131.09
	ZOL14S	399.1119	ND	ND
*T. atroviride* Q710251	Z14S	397.0963	397.0952	317.14, 289.15, 273.16, 175.07, 149.10, 131.09
	ZOL14S	399.1119	ND	ND
*T. asperellum* Q710682	Z14S	397.0963	397.0953	317.14, 289.15, 273.16, 175.07, 149.10, 131.09
	ZOL14S	399.1119	399.1112	319.15, 291.21, 275.20, 257.26, 174.12
*T. virens* Q710925	Z14S	397.0963	397.0946	317.14, 289.15, 273.16, 175.07, 149.10, 131.09
	ZOL14S	399.1119	ND	ND

*ND, Not detected*.

## Discussion

The effective methods to manage mycotoxin contamination include application of antagonistic microbes to prevent mycotoxin production before harvest and using detoxification agents to treat contaminated foodstuffs (Atanasova-Penichon et al., 2016; Perczak et al., 2016; De Saeger and Logrieco, 2017). Due to its potentials to control plant pathogens, the non-toxigenic *Trichoderma* genus has been intensively investigated (Benítez et al., 2004). In the present study, we co-cultured *Trichoderma* isolates with ZEN-producing *F. graminearum* F1 to assess the inhibition and detoxification capacities of tested *Trichoderma* isolates *T. harzianum* Q710613, *T. atroviride* Q710251, *T. asperellum* Q710682 displayed promising antagonistic potentials to control the growth and mycotoxin production of ZEN-producing *F. graminearum* F1. In order to exhaustively access their antagonistic potentials, these *Trichoderma* isolates were dual cultured with other ZEN-producing *Fusarium* species in the later experiment. These antagonists exhibited prominent inhibitory actions on both mycelia spread (Figure S1) and mycotoxin production (Figure S2) of the ZEN-producers. Taken together, our recent progress indicates that the three candidates are potential biological control antagonists to combat toxigenic fungi, which deserve attention and further analysis of their ability to control disease development in field experiments.

**Figure 7:**
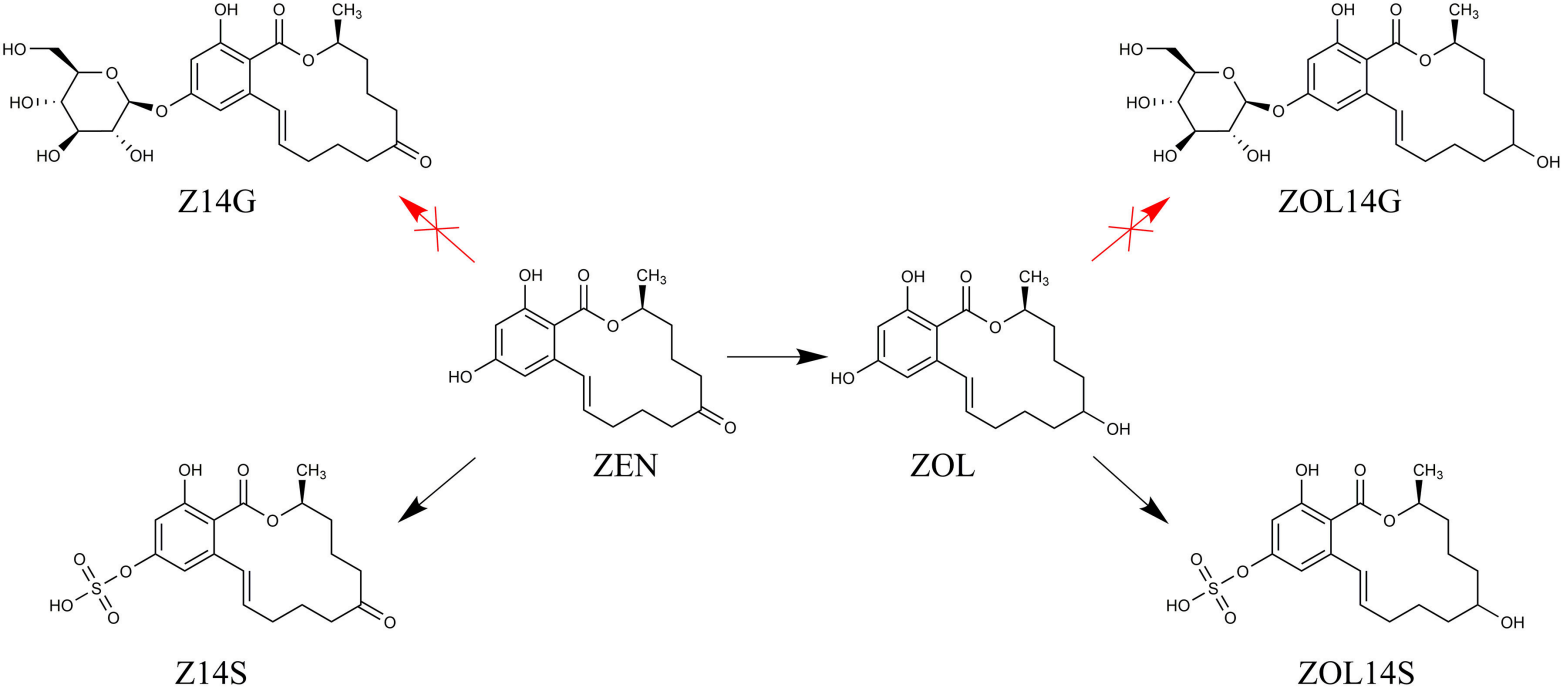
The proposed metabolic detoxification process of mycotoxin ZEN in *Trichoderma* isolates.

Plants possess the capacity to detoxify phytotoxic compounds into low-toxic products after infected by toxigenic fungi. Mycotoxins are toxic xenobiotics for plants, which can be conjugated to polar metabolites in detoxification reactions of plants, generating low-toxic metabolites with structure changed (Berthiller et al., 2016). The detoxification mechanisms of plants against mycotoxins mainly include three phases: transformation phase, conjugation phase and compartmentation phase (Berthiller et al., 2007). Both glycosylation and sulfation are common processes in detoxification reactions of different plants against mycotoxins (Lemmens et al., 2016). It has been showed that DON and ZEN can be bio-transformed into glycosylated and sulfated forms in the detoxification process of plants (Berthiller et al., 2007). The UDP-glucosyltransferase (UGT) capable of converting DON into D3G was firstly identified in *Arabidopsis thaliana* (Poppenberger et al., 2003), and then the first UGT capable of converting ZEN into Z14G was also identified in *Arabidopsis thaliana* (Poppenberger et al., 2006). With regard to DON, our previous work proved that *Trichoderma* spp. possess the ability to metabolize DON into its glycosylated form (Tian et al., 2016b). Consequently, we explored whether *Trichoderma* isolates possess the ability to modify mycotoxin ZEN in this work. Not similar to plants, the tested *Trichoderma* isolates could not bio-transform ZEN into its glycosylated forms, but could convert ZEN into its reduced and sulfated form(s) (Figure 7). Evidence was provided that *Trichoderma* isolates were able to detoxify ZEN via sulfation when competing with ZEN-producing *F. graminearum*.

LC-MS/MS is a useful tool for simultaneous determination of different co-existing mycotoxins when standards are available (Righetti et al., 2016). However, it is still challenging to identify and quantify modified mycotoxins by using LC-MS/MS due to the limited commercial availabilities of modified mycotoxin standards. The HRMS has the advantage of providing accurate ion mass-to-charge that can be used for structure elucidation of compounds in a targeted or untargeted strategy (Righetti et al., 2016), so it has become a promising tool for analyzing the predicted metabolites without standards (De Boevre et al., 2016; Righetti et al., 2016). In our current work, the HRMS was used to obtain accurate mass and fragmentation patterns of analytes, and the sulfated metabolites (Z14S and ZOL14S) produced by *Trichoderma* isolates were discovered. This contributes to further investigations of the defense mechanism of biological control agents against toxigenic fungi.

## Author contributions

AW and YT conceived and designed the experiments; YT, YLT, and ZY performed the experiments and analyzed the data; AW and YT wrote the paper; YL, JC, SD, and MD contributed materials and amended the manuscript.

### Conflict of interest statement

The authors declare that the research was conducted in the absence of any commercial or financial relationships that could be construed as a potential conflict of interest.
