# Supplementary material for: Antagonistic and Detoxification Potentials of Trichoderma Isolates for Control of Zearalenone (ZEN) Producing Fusarium graminearum
Source: Front Microbiol. 2018 Jan 18;8:2710. doi: 10.3389/fmicb.2017.02710 (PMC5778118; doi:10.3389/fmicb.2017.02710)
Supplement: Supplementary file 1 [file Presentation1.PDF]

Supplementary Material:

Antagonistic and detoxification potentials of *Trichoderma* isolates for control of zearalenone (ZEN) producing *Fusarium graminearum*

**Ye Tian [1], Yanglan Tan [1], Zheng Yan [1], Yucai Liao [2], Jie Chen [3], Marthe De Boevre [4], Sarah De Saeger [4], and Aibo Wu [1]*

[1] SIBS-UGENT-SJTU Joint Laboratory of Mycotoxin Research, Key Laboratory of Food Safety Research, Shanghai Institutes for Biological Sciences, University of Chinese Academy of Sciences, Chinese Academy of Sciences, Shanghai 200031, China

[2] College of Plant Science and Technology, Huazhong Agricultural University, Wuhan 430070, China

[3] Department of Resources and Environment Sciences, School of Agriculture and Biology, Shanghai Jiaotong University, 800 Dongchuan Road, Shanghai 200240, China

[4] Laboratory of Food Analysis, Department of Bioanalysis, Faculty of Pharmaceutical Sciences, Ghent University, Ottergemsesteenweg 460, B-9000 Ghent, Belgium

*** Correspondence:**

Aibo Wu

abwu@sibs.ac.cn

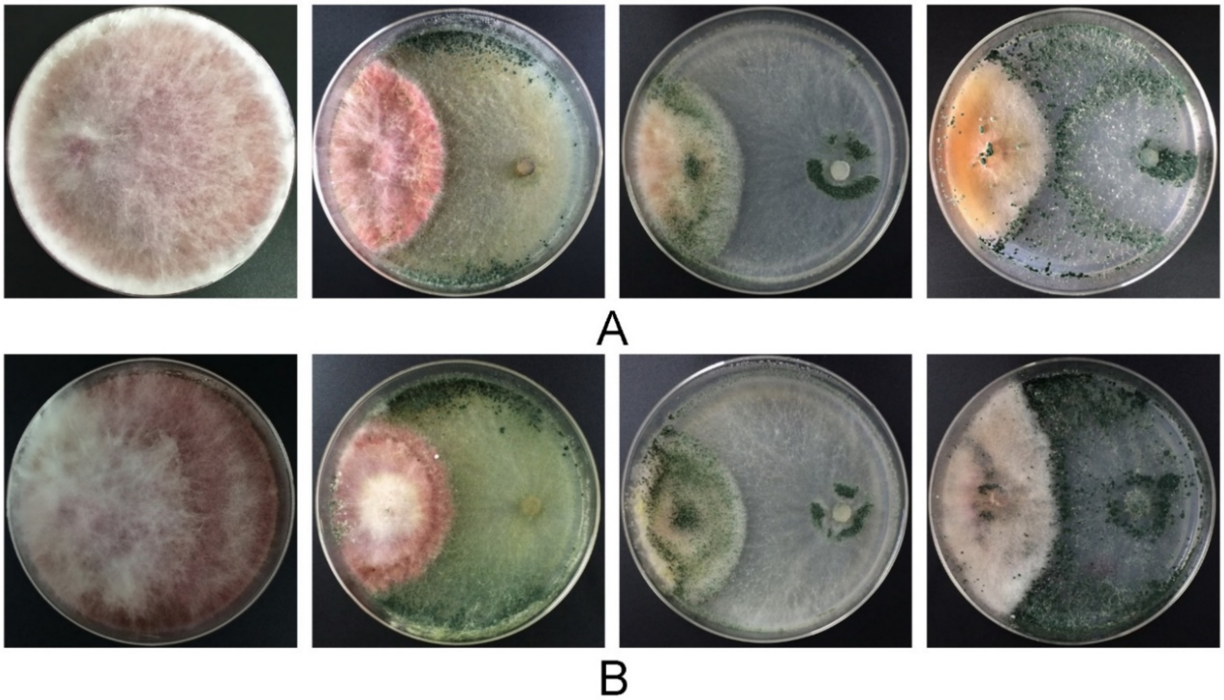

Figure S1. Colony morphology of *F. graminearum* A3 (A) and C1 (B) in co-culture assay after incubation on potato dextrose agar (PDA). From left to right: *F. graminearum* grew alone, and grew against *T. harzianum* Q710613, *T. atroviride* Q710251, and *T. asperellum* Q710682.

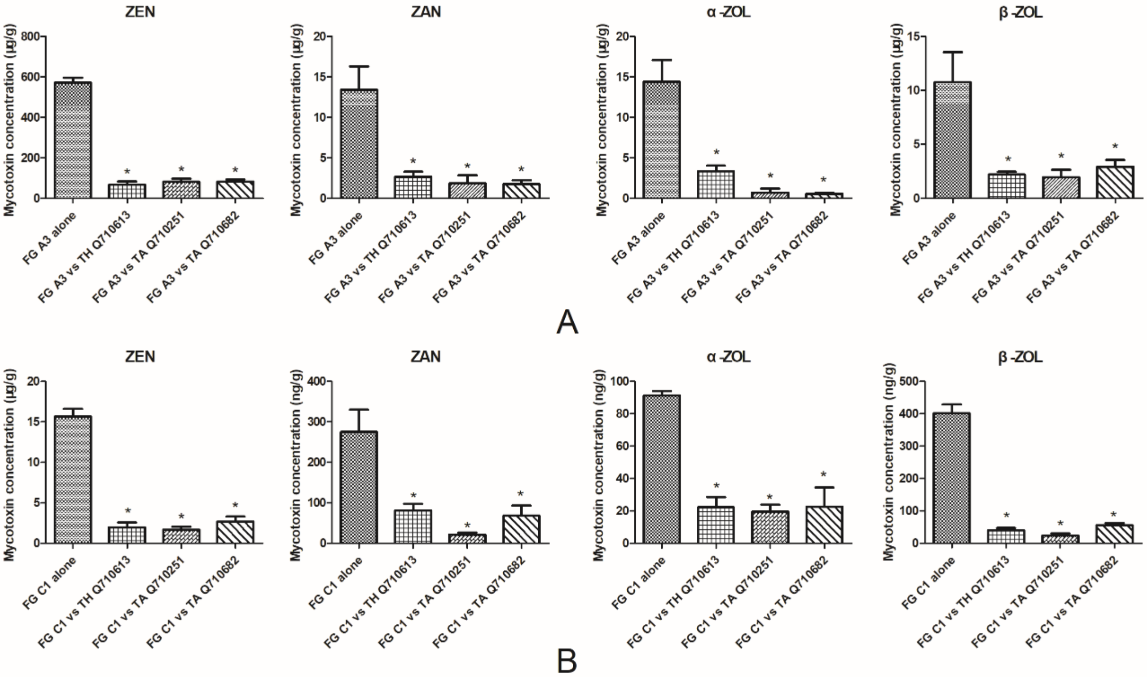

Figure S2. The inhibitory effect of *T. harzianum* Q710613, *T. atroviride* Q710251 and *T. asperellum* Q710682 on mycotoxin production of *F. graminearum* A3 (A) and C1 (B) in co-culture assay. * $P < 0.05$, significantly different from control.
